# Discovery of novel acetylcholinesterase inhibitors through integration of machine learning with genetic algorithm based *in silico* screening approaches

**DOI:** 10.3389/fnins.2022.1007389

**Published:** 2023-03-03

**Authors:** Mohd Imran Khan, Park Taehwan, Yunseong Cho, Marcus Scotti, Renata Priscila Barros de Menezes, Fohad Mabood Husain, Suliman Yousef Alomar, Mohammad Hassan Baig, Jae-June Dong

**Affiliations:** ^1^Department of Family Medicine, Gangnam Severance Hospital, Yonsei University College of Medicine, Seoul, South Korea; ^2^Postgraduate Program in Bioactive Natural and Synthetic Products, Federal University of Paraíba, João Pessoa, Brazil; ^3^Department of Food Science and Nutrition, College of Food and Agriculture Sciences, King Saud University, Riyadh, Saudi Arabia; ^4^Department of Zoology, College of Science, King Saud University, Riyadh, Saudi Arabia

**Keywords:** Alzheimer’s disease, machine learning (ML), virtual screening, molecular dynamics (MD), acetylcholinesterase (AChE)

## Abstract

**Introduction:**

Alzheimer’s disease (AD) is the most studied progressive eurodegenerative disorder, affecting 40–50 million of the global population. This progressive neurodegenerative disease is marked by gradual and irreversible declines in cognitive functions. The unavailability of therapeutic drug candidates restricting/reversing the progression of this dementia has severed the existing challenge. The development of acetylcholinesterase (AChE) inhibitors retains a great research focus for the discovery of an anti-Alzheimer drug.

**Materials and methods:**

This study focused on finding AChE inhibitors by applying the machine learning (ML) predictive modeling approach, which is an integral part of the current drug discovery process. In this study, we have extensively utilized ML and other *in silico* approaches to search for an effective lead molecule against AChE.

**Result and discussion:**

The output of this study helped us to identify some promising AChE inhibitors. The selected compounds performed well at different levels of analysis and may provide a possible pathway for the future design of potent AChE inhibitors.

## Introduction

Alzheimer’s disease (AD), a most common neurodegenerative brain disorder, has affected more than 40–50 million worldwide (Prince et al., 2013, 2015; Wu et al., 2017; GBD 2016 Dementia Collaborators, 2019). This degenerative brain disease is characterized by various clinical signs, such as a decline in cognitive function, and thinking abilities (Perl, 2010; DeTure and Dickson, 2019). First, documented more than a century ago, the etiology of the ailment and pathology of this dementia is still unclear (DeTure and Dickson, 2019). Currently, there is no available treatment to restrict/reverse the progression of this disease. The available therapeutic options only help to improvise the cognitive symptoms (Rasmussen and Langerman, 2019). This most common form of dementia leads to a gradual cognitive impairment, which progresses in the advanced stages (Tarawneh and Holtzman, 2012). The current symptomatic therapeutics include memantine, donepezil, galantamine, and rivastigmine (Yiannopoulou and Papageorgiou, 2020). Among them, memantine is an N-methyl-D-aspartate (NMDA) receptor antagonist (Olivares et al., 2012), while the other three drugs are cholinesterase inhibitors (ChEIs) (Parsons et al., 2013). AD causes a progressive loss of cortical neurons at the central level, especially pyramidal cells involved in higher cognitive functions (Serrano-Pozo et al., 2011; DeTure and Dickson, 2019). From a molecular aspect, it is a multifactorial disease which is characterized by various cellular and molecular processes (Guo et al., 2020). The causes of oxidative stress, neuroinflammation, protein aggregation, cell cycle deregulation, and decreased level of acetylcholine are still unknown (Basile, 2018).

As the “cholinergic hypothesis” suggests, the main factor contributing to AD is the progressive degeneration of cholinergic neurons (Hampel et al., 2019).

One of the most productive treatment approaches is enhancing the acetylcholine (ACh) level, increasing the brain’s cholinergic neurotransmission (Stanciu et al., 2019). On the contrary, acetylcholinesterase (AChE) and butyrylcholinesterase (BChE) hydrolyzed ACh in the brain. Among two cholinesterases (ChEs), AChE is 10^13^ folds more active than BChE, which accounts for almost 80% of ACh hydrolysis (Feng et al., 2017). Therefore the inhibition of AChE becomes a promising therapeutic approach for treating AD.

The development of AChE inhibitors remains a major focus of research for the discovery of AD drug (Mehta et al., 2012; Dos Santos et al., 2018). The use of modern computer-aided drug design (CADD) is one of the most prominent approaches for accelerating the drug discovery process and reducing time (Baig et al., 2016). CADD methods efficiently hunt for the most suitable leads *via* database screening or generate an entirely novel scaffold (Sliwoski et al., 2014; Baig et al., 2018). Furthermore, it analyzes the leads for their interactive power and stability by simulating the binding mode and affinity of the system. The process is comparatively agile, save time, cost, and efforts during the efficient discovery of new therapeutic candidates.

This study utilized three machine learning (ML) approaches and virtual screening to search for effective AChE inhibitors. The combination of ML and molecular docking-based virtual screening was applied to screen the Maybridge compound database. The top-selected compounds were further evaluated using the molecular dynamics (MD) simulation and analyzed for their stability with AChE protein.

## Materials and methods

### Dataset

The molecular dataset with reported experimental IC50 activity against AChE was downloaded from ChEMBL (Gaulton et al., 2012). Molecules with absolute values for biological activity were filtered out. The dataset was further processed to identify the unique molecules having no redundant, incomplete, or chemically errant structures. To build the classification model, all the molecules with IC50 activity values less than or equal to 5 μM were treated as actives, while those having activity values greater than 5 μM were treated as inactives. To preclude the likelihood of data bias, we balanced out the dataset by generating decoys on the directory of useful decoys, enhanced (DUD-E) database (Mysinger et al., 2012). Both active and inactive datasets were initially segregated in smiles format (Kotsiantis et al., 2006). The molecules in the dataset were standardized at physiological pH, and hydrogen atoms were added. The molecules were then converted into 3D-structure using the Open Babel software (version 3.0.0) (O’Boyle et al., 2011).

### Descriptor generation

The standardized 3D MDL MOLfiles of the selected dataset were used for molecular descriptor generation. An open-source PaDEL software was utilized to calculate molecular descriptors and fingerprints (Yap, 2011). A total of 1,444 1D and 2D descriptors, 431 3D descriptors, and 881 PubChem substructure fingerprints were estimated. Statistical analyses like ANOVA, the Kruskal test, and a chi-square test in R packages were used to reduce the chances of overfitting the descriptor feature selection.

### Machine learning

The feature selection, ML model generation, and validation were performed using Weka version 3.8.4 (Bouckaert et al., 2010). The feature selection was done using CFS subset Eval (Attribute Evaluator); subsequently, the relevant features were picked up by Best First search.

The entire dataset containing active and inactive categories was split into train and test sets in a ratio of 30:70 randomly. We used three different algorithms, namely, random forest (Schonlau and Zou, 2020), support vector machines (SVM) (Chang and Lin, 2011), and multilayer perceptron (MLP) (Taud and Mas, 2018) for building ML models.

All the models built were validated for their performance using a test group of molecules. The models were ranked according to the Matthews correlation coefficient (MCC) (Chicco and Jurman, 2020), the best-performing algorithm was selected. Subsequently, the selected model was further used for screening the Maybridge database. The database was also preprocessed to contain the descriptor information required for screening purposes. Finally, we filtered out the molecules predicted to be over 90% active.

### Molecular docking

The structure of human AChE in complex with Donepezil was extracted from the RCSB, PDB ID: 4EY7 (Cheung et al., 2012). Donepezil was redocked within the binding site of AChE using the CCDC Gold software package (Verdonk et al., 2003). The binding orientation of the redock and crystal confirmation of Donepezil within the active site of AChE was compared. The molecules screened from the ML were filtered further against AChE using the molecular docking approach. The top molecules were selected based on their piecewise linear potential (PLP) fitness scores.

### Molecular dynamics simulations

The top five high-scoring molecules in complex with AChE were subjected to MD simulation studies to evaluate their binding stability. GROMACS (version 2020.04) was used to perform the MD simulation of all the selected complexes (Abraham et al., 2015). A cubic box with margin radius of 10 Å was selected for solvation with TIP3P solvent model. The system was treated with CHARMM27 force field (Vanommeslaeghe et al., 2010). Ligand topology was generated at the SwissParam web server (Zoete et al., 2011). The system was neutralized using sodium ions and chloride ions. The complex box was energy minimized using the steepest descent minimization algorithm, keeping at constant force for 50,000 steps. Isothermal and isochoric equilibration was done using particle-mesh Ewald for long-range electrostatics. Isothermal and isobaric equilibrations were carried out using particle-mesh Ewald followed by a production run of 500 ns. The biophysical parameters for proteins and ligands were calculated using the initial starting structure as a reference frame.

## Results

### Data processing and model building

A total of 2,538 unique molecules reported against AChE were selected from the ChEMBL database. The dataset was segregated into 2,037 active (IC50 ≤ 5 μM) and 501 inactive molecules (IC50 > 5 μM). Imbalance data can seed a biased input data load, leading to a faulty model and therefore, 1,536 DUD-E inactive decoys were generated to rule out experimental parti pris. The PaDEL software created 1,024 MACCS and PubChem physicochemical property fingerprints for 2,037 active and an equal number of inactive small molecules as an input data for modeling.

The ML quantitative structure-activity relationship (QSAR) model generated using three different approaches- namely, random forest, support vector machine, and multi-layer perceptron. The characteristic validation terms for comparing different models in terms of their predictive power and robustness are given in Table 1. The random forest model displayed the best performance for accuracy, precision, recall, F-measure, MCC, and receiver operating characteristic (ROC) area among the three models. The Mathews coefficient of correlation value for the RF model was 0.85, the highest among the three models.

**TABLE 1:** Evaluation of machine learning models (RF, SVM, and MLP).

	Accuracy	Precision	Recall	F-measure	MCC	ROC area
Random forest	94.1056	0.942	0.941	0.941	0.846	0.976
Support vector machine	90.0532	0.900	0.901	0.900	0.734	0.863
Multi-layer perceptron	93.1232	0.932	0.931	0.932	0.820	0.960

**FIGURE 1:**
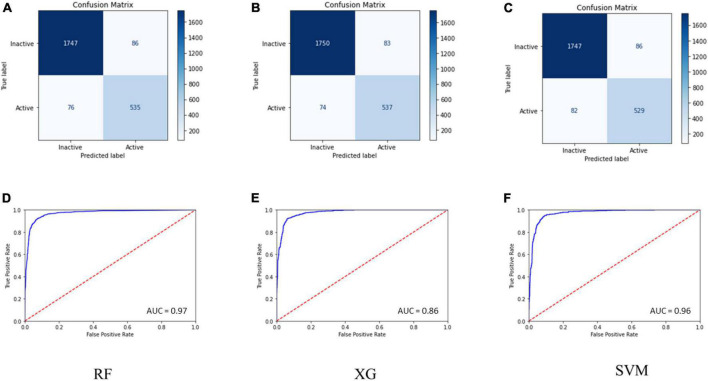
**(A–C)** Confusion matrix for the RF, SVM, and MLP models. **(D–F)** ROC curve for the performance of binary class models.

Internal 10X cross-validation was performed, and the data regarding true positives, true negatives, false positives, and false negatives for the confusion matrix, which is reflective of the classifier’s quality parameters, are illustrated in Figures 1A–C.

The ROC plot was generated for the truly predicted positive classes among all actual positive classes and the incorrectly predicted negative classes among all actual negatives. The RF binary classifier’s performance was found to have the highest area under the ROC curve (AUC) of 0.97 (Figures 1D–F). Collectively, the RF model achieved the best for all the validation terms; hence, it was selected for further ligand-based virtual screenings studies.

### Ligand-based virtual screening

The model generated was used to screen the Maybridge library of 51,775 molecules. The library consists of highly active molecules, and we intend to identify novel and potent AChE inhibitors. The ML screening eventually spotted 922 molecules with a prediction score of above 90%.

### Molecular docking

**FIGURE 2:**
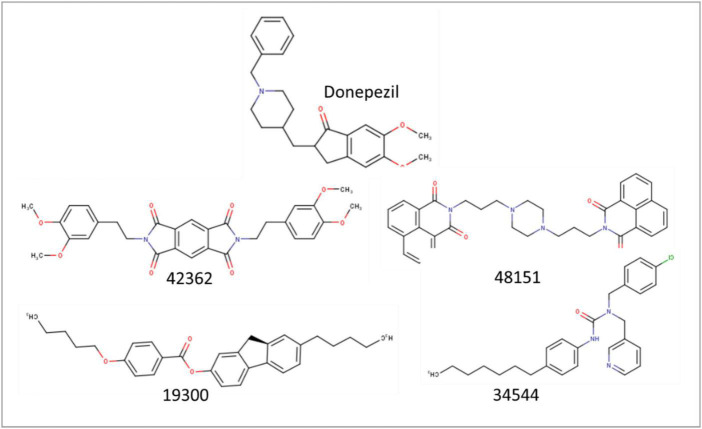
Stick representation of the 2-dimensional structure of the top four selected molecules in ligand-based virtual screening.

The selected 922 molecules were further evaluated using the docking-based virtual screening approach. These molecules were docked using the CCDC GOLD software within the active site of the AChE enzyme to evaluate the binding affinity along with Donepezil, an approved AChE inhibitor. The top four scoring molecules, viz. 42362 (Compound 1), 48151 (Compound 2), 34544 (Compound 3), and 19300 (Compound 4), were selected based on their PLP Fitness score (Table 2 and Figure 2).

**TABLE 2:** The ChemPLP binding scores of the reference inhibitor drug and the best predicted active molecules.

Molecules	ChemPLP score
Donepezil	−102.7
Compound 1 (42362)	−123.99
Compound 2 (48151)	−122.25
Compound 3 (34544)	−121.32
Compound 4 (19300)	−119.49

All four identified molecules had an elongated chain-like structure with different moieties attached to the central frame. We further performed MD simulation, and free energy calculation to establish their stability within the protein’s active site.

The selected molecules showed better docking scores than the reference drug (Donepezil), indicating their higher binding affinity. The selected molecules showed a strong interaction with the AChE active site residues. The results are promising and suggest an appreciable probability of them acting as a potential AChE inhibitor molecule transcending the current therapeutic drugs.

Compound 1 had the highest interaction affinity score of −123.99, followed by Compound 2, Compound 3, and Compound 4. These hits are symmetrical, having a similar heterocyclic aromatic nucleus in their chemical structures. A known challenge to all inhibitors for neurological diseases such as AD is the ability to cross the blood–brain barrier to inhibit AChE (Michalska et al., 2000). This makes it an essential characteristic requirement for any AD drug candidate (Michalska et al., 2000). Compound 2 has a naphthalimide moiety that confers a unique physicochemical characteristic that allows it to cross biological membranes easily.

**FIGURE 3:**
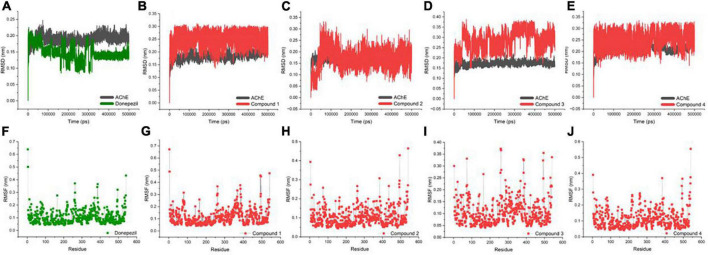
Biophysical simulation analysis of the complexes. Root-mean-square deviation (RMSD) plot of AChE in complex with **(A)** Donepezil, **(B)** Compound 1, **(C)** Compound 2, **(D)** Compound 3, and **(E)** Compound 4; RMSF plot of AChE in complex with **(F)** Donepezil, **(G)** Compound 1, **(H)** Compound 2, **(I)** Compound 3, and **(J)** Compound 4.

An MD trajectory analysis was also performed for the four identified molecules and the reference inhibitor (Donepezil) to evaluate the stability and consistent interactions with the AChE protein. The root-mean-square deviation (RMSD) was calculated separately for the ligand and the protein for the entire dynamics time to analyze the thermal fluctuations (Figures 3A–E). The mean value of RMSD was approximately 0.2 Å for the AChE-Donepezil, and its value decreased to ∼0.17 Å in the later part of the dynamics. Compound 3 was slightly unstable until 100 ns, which was later stabilized with high amplitude (2 Å) for the remaining time. For Compounds 1, 2, and 4, the RMSD consistently remained stable throughout the simulation with an average RMSD of 0.23 Å.

Residues with higher atomic variations impart instability, making the ligand diffuse away from its initial binding site. We identified root-mean-square fluctuations (RMSFs) for the proteins in all the complexes (Figures 3F–J). The residual RMSF values were found to be similar for all the complexes in the study. Protein residues 75, 76, 121, 292, 294, 295, 339, and 340 positioned in the interacting vicinity of Donepezil have no significant fluctuation.

Furthermore, we applied the molecular mechanics Poisson-Boltzmann surface area (MMPBSA) approach to determine Gibbs free energy of binding (Genheden and Ryde, 2015). It is a valuable equation to understand physiological interfaces, describing the distribution of electrical potential in solution in the normal direction for a charged surface (Yap, 2011). Table 3 shows the binding free energy value of the selected compounds using the simulation trajectories.

**TABLE 3:** Variations of the total Gibbs free energy of the molecules analyzed in the study of molecular dynamics.

Molecules	Poisson Boltzmann–ΔG_TOTAL_ (differences: complex–receptor–ligand)
Donepezil	−27.73 ± 4.03
Compound 1	−30.97 ± 2.41
Compound 2	−50.44 ± 1.06
Compound 3	−25.21 ± 1.96
Compound 4	−23.50 ± 2.47

We found that Donepezil has a ΔG value of −27.73 ± 4.03, which is higher than Compounds 1 and 2. The energy attained a constant value after ∼120,000 ps of dynamics. Molecule 48151 displayed the lowest ΔG value (−50.44 ± 1.06) transcending Donepezil.

### Principal component analysis

**FIGURE 4:**
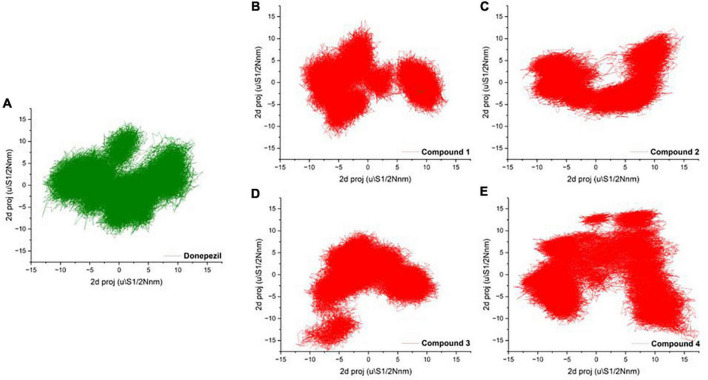
**(A–D)** 2D projection of the trajectory allocation for the motion of the proteins in complex with **(A)** Donepezil, **(B)** Compound 1, **(C)** Compound 1, **(D)** Compound 3, and **(E)** Compound 4 in the essential subspace.

To mark out the significant atomic motions in proteins, we carried out principal component analysis (PCA) on the simulation trajectories (David and Jacobs, 2014; Khan et al., 2021). We identified and evaluated the most prominent eigenvectors and analyzed the average trajectory (Mazanetz et al., 2014). We extracted the projection from the top six eigenvectors for the PCA of AChE bound with Donepezil and four high-scoring hits. The trajectory suggested different atomic motions during the simulation. The 2D projection of the trajectories in the essential subspace is projected in Figures 4A–E. The results showed that Compounds 1 and 2 bound structure of AChE occupied a common conformational space, indicating higher complex structural stability.

**FIGURE 5:**
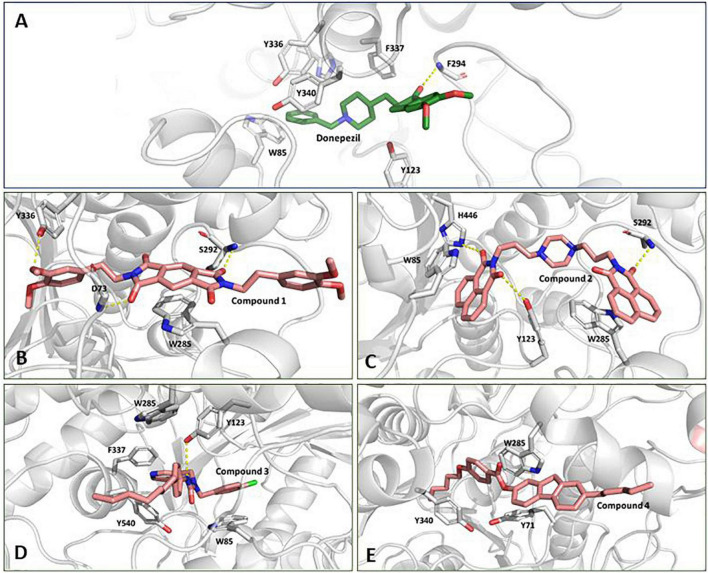
Conformation of **(A)** Donepezil, **(B)** Compound 1, **(C)** Compound 2, **(D)** Compound 3, and **(E)** Compound 4 in the AChE protein active site (ribbon representation) showing intermolecular interaction.

We inspected the trajectories extracted by averaging the ensembles after attaining stability to get more insight into the binding interaction (Figures 5A–E). Compound 1 has a central benzo di-succinimide moiety. It is identically attached to two dimethylethylbenzene groups on both sides. The molecule exhibited some crucial stabilizing interactions with the amino acid residues on the active site of AChE. The oxygen atom of the benzo di-succinimide ring consistently formed hydrogen bond interactions with S292 and D73 residues. In addition to it, the hydroxyl group of the dimethylethylbenzene interacted through a hydrogen bond with Y336. The benzyl ring in the central moiety was found to interact with Pi-Pi stacking interaction to the aromatic ring of the W285 on the P-site of the AChE protein. The molecule showed an appreciable binding affinity to the AChE protein with binding free energy better than Donepezil (Figure 5B). Compound 2 has a centrally located piperazine ring. The moiety is attached to the fused benzopiperidine-dione from both sides at the amino position *via* an ethyl bridge. The oxygen atom of the benzopiperidine-dione moiety was found to interact with imidazole side chain nitrogen of H446 and with hydroxyl group of Y123. While the second fuse benzopiperidine-dione moiety had hydrogen bond interaction with the backbone nitrogen of S292. These bonds were found consistent throughout the simulation period (Figure 5C).

Compound 3 has a central inorganic urea which has its amine group attached to the aromatic benzylic pyridine and benzylic chlorobenzene one one side. While the second amide group on the central moiety is attached to the benzene group with a long and heptane chain attached. The molecule is bonded to the active site residues predominantly *via* sandwich pi stacking type hydrophobic interaction with W85. A hydrogen bond between the hydroxyl group of Y123 and the amide nitrogen atom on the central core of the molecule was very consistent throughout the simulation period (Figure 5D). Compound 4 consists of a 4-pentoxy benzoic acid attached to the fluorene group. A hydrophobic n-pentane chain was attached to the fluorene moiety. The molecule has a ΔG value, relatively higher than the reference molecule, Donepezil. The molecule was found to fit deep along the pocket with its long pentane chain lying along the hydrophobic patch at the end of the protein binding pocket. It was found to interact with Y340, W285, and Y71 residues (Figure 5E). The molecule possessed a long aromatic chain-like structure and due to this structural characteristic, it developed dominant Pi, Pi-Pi stacked, Pi-Sigma, and Pi-alkyl-type interactions with the protein.

## Discussion

Alzheimer’s disease is a chronic progressive neurodegenerative disorder and one of the most studied diseases (Kirkitadze et al., 2002). Prevailing AD therapeutics work to relieve symptoms and delay the progression (Duraes et al., 2018). In this study, we targeted AChE to modulate the ACh level in the brain.

The protein has four isoforms with almost similar sequences. The shortest one is 526 amino acids long, while the other three have 603, 614, and 617 amino acids, respectively. The mature part of the protein starts after 31 signal peptides. The 3D structure is composed of a beta sheet made up of 12 beta strands surrounded by alpha helices on both sides. The structure is compact. The active site is deep and surrounded by residues with stable regular secondary structure orientation. Y103, W117, G152, Y155, E233, S234, W317, S324, V325, F326, Y368, F369, Y372, H478, and G479 are active site residues dictating the binding interactions at the active site. A deep tunnel-shaped binding pocket is acidic in nature. Donepezil, an AChE inhibitor, is a widely used drug in treating AD and its mechanism of action is very clear (Shintani and Uchida, 1997). We identified AChE inhibitors through the ML predictive modeling approach, an integral part of the novel drug discovery process to prevent cholinergic neurons from degeneration (Vamathevan et al., 2019; Dara et al., 2022). Ligand-based drug design methods that employ quantitative descriptor information of a known chemical structure with an identified biological activity to form an ML model are cost-effective method to develop novel lead molecules (Baig et al., 2016; Neves et al., 2018; Selvaraj et al., 2021).

We used three different ML algorithms (random forest, support vector machine, and multi-layer perceptron) to build classification models. Y-scrambling test, k-fold cross-validation, and k-holdout validation were performed on the activity dataset to rule out the fortuitous results during modeling. The random forest model was the best-identified method against the AChE enzyme. The statistical parameters such as accuracy, precision, recall, F-measure, MCC, and ROC values were highest for the RF model. The model was used to screen the Maybridge library, and we identified the top four scoring molecules. These ML leads were then evaluated for tier-two validation. The identified molecules and the reference drug molecule were subjected to molecular docking and MD simulation to compare their potency against the target enzyme. The PLP score for binding to AChE for Compound 1 (−123.99), Compound 2 (−122.25), Compound 3 (−121.32), and Compound 4 (−119.49) was higher as compared to the known drug, Donepezil (−102.7).

The docked pose and the crystal orientation showed that the benzylic ring of Donepezil was involved in a hydrophobic interaction with the amino acid residue W85, Y123, F337, Y340, and Y336 (Figure 5A). The indanone ring showed a pi-stacking-type interaction with the residue Trp 286. The RMSD values for all four molecules throughout the simulation remained stable, with their values around 2 Å, similar to Donepezil, indicating that these compounds could be used as potential AChE inhibitors.

The PCA showed no significant changes among the complexes. Compound 4 showed a relatively high thermal motion with states scattered in the broader conformational space. Protein secondary structures were found to be stable without any major conformation transition. These findings suggest uniform packing and stability of the complexes.

The Gibbs free energy calculated is in agreement with the previous results. Compounds 1, 3, and 4 have energies comparable to the reference drug, while Compound 2 reflected the binding free energy ∼1.8 times higher than Donepezil.

In this study, we proposed the four highly potent molecules against AChE. The selected compounds performed well at different levels of analysis in the scope of our findings. A structured *in vitro* experiment can help confirm its potency in inhibiting the AChE protein.

## Conclusion

Alzheimer’s disease, a multifaceted neurodegenerative disease with no available cure, has presented significant challenges. Despite great scientific efforts, no major success has been made in successfully treating this neurodegenerative disorder. In this study, we have employed the ML approach to deduce a ligand-based mathematical model. The model was trained and tested on the experimental biological activity bioassay data. Subsequently, the best model was employed to screen the Maybridge molecular database for potent hits against AChE. This study identified four high-performing AChE inhibitors. These molecules showed more improved binding affinity as compared to the known drug molecule. Furthermore, we proposed that the identified molecules can act as a highly active starting scaffold which may be further used and studied for developing putative anti-AD drugs.

## Data availability statement

The raw data supporting the conclusions of this article will be made available by the authors, without undue reservation.

## Author contributions

MB and J-JD: conceptualization. MK, PT, YC, MS, RP, FH, and SA: methodology. MB, J-JD, MS, SA, and FH: formal analysis and investigation. MS, RP, MB, FH, and J-JD: writing—original draft preparation. All authors contributed to the article and approved the submitted version.
